# Pre-Retinal Hemorrhage on Point-of-Care Ultrasound

**DOI:** 10.5811/cpcem.2018.6.38459

**Published:** 2018-07-18

**Authors:** Krystal Garcia, Julian Jakubowski, Linette Archer

**Affiliations:** Marietta Memorial Hospital, Department of Emergency Medicine, Marietta, Ohio

## CASE PRESENTATION

A 30-year-old male presented to the emergency department (ED) with sudden, painless, decreased vision in the left eye after an episode of severe vomiting. He noted a gray area in the center of his vision and was only able to distinguish objects’ outlines with the affected eye. His visual acuity was 20/200 in the left eye vs. 20/50 in the right. Intraocular pressures were 18 millimeters of mercury (mmHg) in the left eye and 16 mmHg in the right eye. Point-of-care ultrasound (POCUS) (Image, Video) showed findings consistent with retinal pathology and hemorrhage. No further workup was obtained in the ED. Ophthalmology was consulted with the ultimate diagnosis of pre-retinal hemorrhage due to Valsalva action.

## DIAGNOSIS

Valsalva retinopathy is a rare entity, most commonly presenting as pre-retinal hemorrhage either bilaterally or unilaterally, which to these authors’ knowledge has not previously been identified on POCUS in an ED. It is normally self-limited with a favorable prognosis and resolution over several months.^1^,^2^ The mechanism of Valsalva retinopathy is due to a sudden increased thoracic/intra-abdominal pressure leading to a rapid increase in intraocular venous pressure and spontaneous rupture of capillaries.^2^ Aside from conservative management, other treatments are available for resolution of pre-retinal hemorrhage including the following: pneumatic displacement of hemorrhage with yttrium-aluminum-garnet laser, tissue plasminogen activator, vitrectomy, or the less-invasive injection of intravitreal ranibizumab (anti-vascular endothelial growth factor).^2^

Use of POCUS allowed for quick diagnosis, consultation, and disposition without the need for extensive or expensive testing. POCUS findings seen in image and video revealed evidence of retinopathy, and subsequent optic imaging confirmed pre-retinal hemorrhage. This furthers the evidence of the utility of POCUS in differentiating pathology and obtaining quick consultation as needed.^3^

Documented patient informed consent and/or Institutional Review Board approval has been obtained and filed for publication of this case report.

**CPC-EM Capsule**

What do we already know about this clinical entity?

Valsalva-induced pre-retinal hemorrhage is a known entity in ophthalmology but is a rare finding that would not normally be diagnosed in the emergency department (ED).

What is the major impact of the image(s)?

The images allow emergency physicians to learn the importance of being able to obtain and recognize point-of-care ultrasound (US) images at bedside.

How might this improve emergency medicine practice?

This report shows the ever-increasing utility of point-of-care US not only as a tool for procedures, but also for diagnosis in the ED.

## Supplementary Information

Video Pre-retinal hemorrhage.

## Figures and Tables

**Image:**
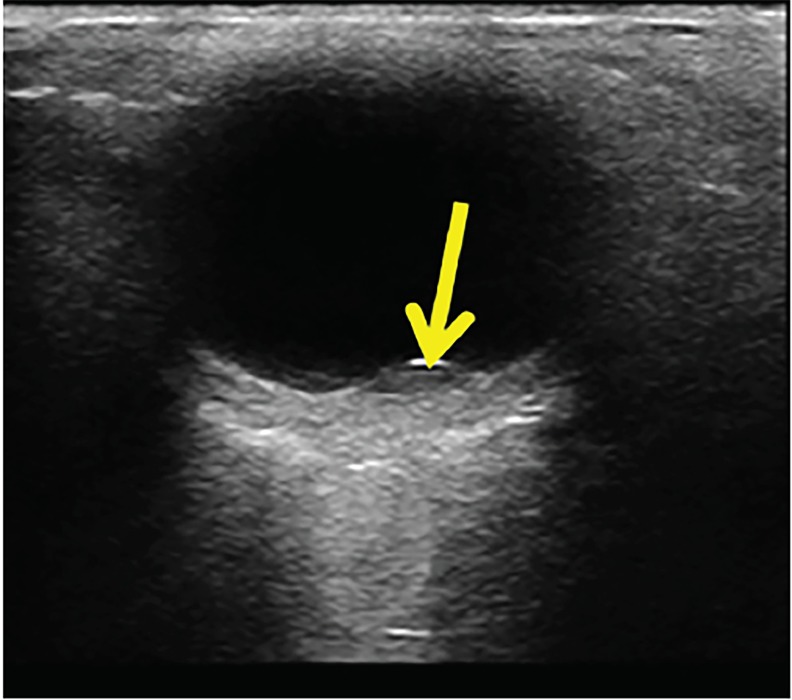
Point-of-care ultrasound demonstrating a pre-retinal hemorrhage (arrow).
